# Algorithms designed for compressed-gene-data transformation among gene banks with different references

**DOI:** 10.1186/s12859-018-2230-2

**Published:** 2018-06-18

**Authors:** Qiuming Luo, Chao Guo, Yi Jun Zhang, Ye Cai, Gang Liu

**Affiliations:** NHPCC/Guangdong Key Laboratory of popular HPC and College of Computer Science and Software Engineering, Shenzhen University, Shenzhen, 518060 China

**Keywords:** Reference-based compression, DNA sequence compression, Gene data transformation

## Abstract

**Background:**

With the reduction of gene sequencing cost and demand for emerging technologies such as precision medical treatment and deep learning in genome, it is an era of gene data outbreaks today. How to store, transmit and analyze these data has become a hotspot in the current research. Now the compression algorithm based on reference is widely used due to its high compression ratio. There exists a big problem that the data from different gene banks can’t merge directly and share information efficiently, because these data are usually compressed with different references. The traditional workflow is decompression-and-recompression, which is too simple and time-consuming. We should improve it and speed it up.

**Results:**

In this paper, we focus on this problem and propose a set of transformation algorithms to cope with it. We will 1) analyze some different compression algorithms to find the similarities and the differences among all of them, 2) come up with a naïve method named TDM for data transformation between difference gene banks and finally 3) optimize former method TDM and propose the method named TPI and the method named TGI. A number of experiment result proved that the three algorithms we proposed are an order of magnitude faster than traditional decompression-and-recompression workflow.

**Conclusions:**

Firstly, the three algorithms we proposed all have good performance in terms of time. Secondly, they have their own different advantages faced with different dataset or situations. TDM and TPI are more suitable for small-scale gene data transformation, while TGI is more suitable for large-scale gene data transformation.

## Background

With the development of the sequencing technologies, the cost for sequencing has become lower and lower, while the speed of sequencing has become faster and faster. As a result, we will find that the gene data from various species is experiencing an explosive growth and we have been in an era of gene big data. Human Genome Project [1], launched in 1990, using the first generation of gene sequencing technology, took 13 years and cost 3 billion dollars, finally completed by a number of scientists from multiple countries around the world. Now Illumina’s latest gene sequencing platform, the HiSeq X Ten system, requires only $1000 to sequence the whole gene of a single person and can complete sequencing of more than 18,000 human genomes throughout a year [2, 3]. Nowadays, more and more gene projects are set up [4–8], so gene data will continue to accumulate expansion. Facing with such a large amount of data, how to store, transmit and analyze will be a big problem for researchers [9].

For dealing with the problem of storing, gene compression is an essential mean [10, 11]. So far, there are some gene compression algorithms which are effective have been proposed. Generally, these methods are divided into two categories depending on whether they are based on reference or not. These algorithms based on non-reference, such as BIND [12], DNACompress [13], GeNML [14], XM [15] and POMA [16], could not handle these gene data that is going through explosive growth effectively. On the contrary, algorithms based on reference are the state-of-the-art approach, because they exploit the similarity between sequences (e.g., humans have at least 99.5% of gene similarity and the similarity between gorilla and human is as high as 99% [17]). With the algorithms based on reference, many countries has built their own gene bank, such as NCBI (National Center for Biotechnology Information), The EMBL (European Molecular Biology Laboratory), DDBJ (DNA Data Bank of Japan) and CNGB (China National Gene Bank). However, there exists a big obstacle for sharing information among all of these institutions, because these institutions may select different sequences as the reference. The traditional workflow is decompression-and-recompression workflow, which means that we should decompress the dataset that is compressed with one reference, and then compress it with another reference. Obviously, it is not direct and time-consuming.

Focusing on the problem, in this article, we propose a set of transformation algorithms to cope with it. The traditional workflow just exploits the similarity between dataset and reference, but it ignores the similarity between references. Our new transformation algorithms exploit the similarity of references to avoid the traditional decompression-and-recompression workflow. They simplify the original workflow to reduce large amounts of time.

### Related work

Due to the traditional compression tools and algorithms based on non-reference not dealing with gene data effectively, we will pay our attentions on the algorithms based on reference.

The main concept of referential compression is, given a to-be-compressed sequence and a reference, writing an output file containing only the differences between the two input sequences. Generally, there is three steps in the framework:

1. Build an index for the given reference;
2. Search the corresponding position of to-be-compressed sequence in the reference, using the index,
3. Finally encode the to-be-compressed sequence with the information from step 2 and then encode the preliminary results to produce the final file.

Though a great deal of efficient referential compression algorithms have been proposed [18–22], we just select FRESCO [23], ERGC [24] and ODI [25] as typical tools. We will discuss these tools in detail next.

FRESCO is a referential compression algorithm proposed by Sebastian et al. in 2013. Ignoring the time of building index, it is the fastest compression algorithm while its compression ratio is pretty good. It uses a hash table to index the complete reference genome. Its value is the position where k-mer is found in the reference and its key is the hash value calculated by each k-mer in the reference. With the index, FRESCO use the same hash function to get the key of each non-overlapping k-mer in the to-be-compressed sequence, and then search them with the index. A successful lookup returns a list of positions where the k-mer can be found in the reference. For each match, we extend the match through direct comparison with the reference and pick out the longest match. If the length of the longest match is longer than the threshold, a new entry which record a tuple containing position PF and the length of the match LEN is created in the result and the next lookup on the table will use the k-mer starting on position PF + LEN. If not, the base pair on position PF + 1 is set as a difference between matches and a new lookup will be made using the k-mer starting on position PF + 1. This method will repeat until the entire to-be-compressed sequence is processed.

FRESCO need too much time to build the index structure, which will caused that if the gene data required to compressed is small-scale, the time for building index is far more than the time for compression. ODI algorithm exploits the fact that the similarity rate of the homologous species at the corresponding position is far higher than that at other positions to build partial index structure. There are four main methods in the comparison compression process (RP represent the pointer to the reference and CP represent the pointer to the to-be-compressed sequence):

- SNP detection algorithm. Match segments from the reference and the to-be-compressed sequences directly.
- SNP test. Test if the previous match ended in a Single Nucleotide Polymorphism.
- Brute-force search. Execute a brute-force search for a match within δ base pairs.
- Index lookup. Index *∆* base pairs from the reference starting on the current RP and perform one table lookup (using the k-mer starting at CP), just like FRESCO. If the lookup returns more than one entry, we choose the one most close to the RP.

Generally, we always build index with the whole reference sequence, making the index structure is too large. In the process of searching, the positions of most match is close to the target sequence’s corresponding position in the reference sequence. ERGC employs a divide and conquer strategy. At first ERGC divides the entire reference and target genomes into parts of equal sizes and processes each pair of parts sequentially. For each part, ERGC build a hash index structure with the length of K which is decided by the length of the part and is the length of k-mer at the same time. If lookup return an empty list, ERGC rebuild the index structure with a factor less than K and do search again. Then ERGC calculate the edit distance of the mismatched area and decide whether the edit distance or character information need to be recorded into the result. Finally, ERGC compress the stored information using delta encoding [26] and encode the stored information using PPMD encoder [27].

Referential compression algorithm is mainly used to compress sequences which are highly similar to the reference, recording the same and the differences between to-be-compressed sequences and the reference. Obviously, compression and decompression are very dependent on the reference. For the homologous species in different gene banks, the compressed data could not share directly, because they may use different sequence as reference in the compression process. As a result, when we need to merge the data from two different gene banks based on different references, the traditional workflow is decompression-and- recompression, which is a waste of time. Given this, we should propose a more succinct algorithm to make the data transformation from one gene bank to another faster. At the same time, we can know that these compression algorithms are so different and each of them has its own peculiarity, which we should take into account when we design our own algorithms.

## Methods

### Framework

According to the features of referential compression algorithms, it is obvious that with the compressed data of target sequence and the reference, we can get the distribution of the target sequence on the reference. Given a compressed dataset compressed with the reference Ref1, our goal is to get the compressed dataset compressed with the reference Ref2, which means we replace Ref1 with Ref2 for the dataset. The traditional process is decompressing the dataset with Ref1 and then compressing the former result with Ref2. However, the work above just exploit the similarity between the sequences in dataset and Ref1 and the similarity between the sequences in dataset and Ref2, ignoring the similarity between Ref1 and Ref2. This is right where we can improve it.

**Fig. 1:**
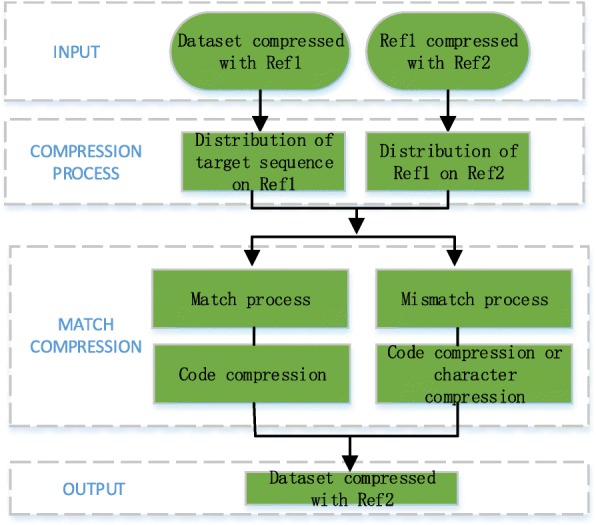
Through this transformation framework, we get the distribution of target sequence on Ref1 and the distribution of Ref1 on Ref2 when processing the compressed data. Then we can make full use of the similarity between Ref1 and Ref2 to make transformation faster

To make full use of the similarity between Ref1 and Ref2, what we do is to compress Ref1 with Ref2, getting the distribution of Ref1 on Ref2. Supposed that we have get the distribution of the sequences in dataset on Ref1 and the distribution of Ref1 on Ref2, we can easily get the distribution of the sequences in dataset on Ref2 through transformation process. The framework is shown below. Exploiting the similarity between Ref1 and Ref2 to transform compressed data, it can avoid the process of decompression-and-recompression to save a lot of time Fig. 1.

### Data process

To obtain the distribution of one sequence on another sequence, we must know how to encode the match and the mismatch. For the match, we record its position and length, while for the mismatch we record its character.

**Fig. 2:**
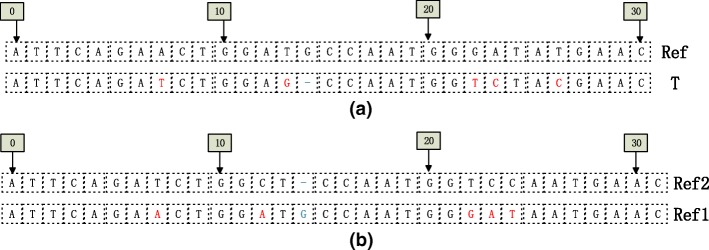
In (**a**), T(target sequence) can be encoded with Ref(reference) as (0.6)(7,7,T)(8,12)(13,14,G)(15,21)(22,23,TC)(24,25)(26,26,C)(27,30). In (**b**), Ref2(references) can be encoded with Ref1(reference1) as (0,6,0,6)(7,7,7,7,A)(8,11,8,11)(12,12,12,12,A)(13,13,13,13)(14,14,13,13,G)(15,21,14,20)(22,24,21,23,GAT)(25,30,24,29)

Like the Fig. 2 showing, the target sequence will be encoded through reference as triples, which is like (start_pos, end_pos, misstr).

Having known how to encode target sequence with reference, we also need to know how to encode a reference with another reference in order to figure out the distribution between two references. Given that there exists two references Ref1 and Ref2. We can encoded Ref2 with Ref as tuples, which is like (Ref2start_pos,Ref2end_pos,Ref1start_pos,Ref2end_pos, misstr).

### TDM

**Fig. 3:**
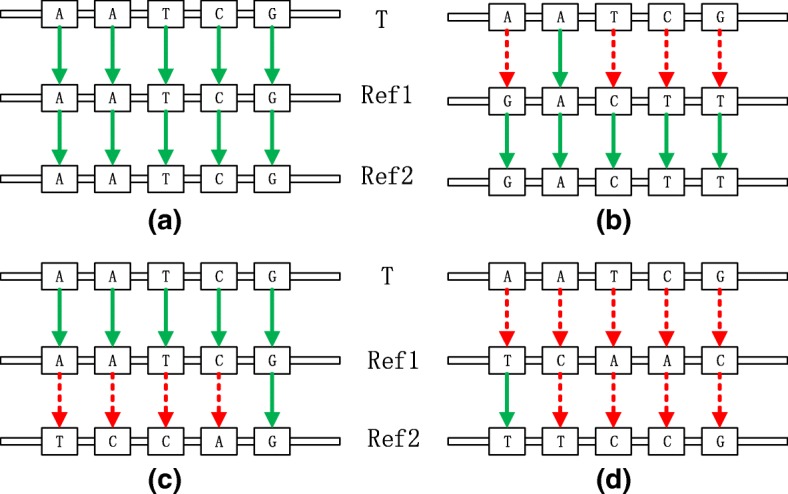
Case (**a**) means that in this area, T is same to Ref1 and Ref1 is same to Ref2, so T is same to Ref2. Case (**b**) means that in this area, T is different with Ref1 but Ref1 is same to Ref2, so T is different with Ref2. Case (**c**) means that in this area, T is same to Ref1 but Ref1 is different with Ref2, so T is different with Ref2. Case (**d**) means that in this area, T is different with Ref1 and Ref1 is different with Ref2, so we could not figure out the relationship between T and Ref2

There exist a target sequence T and two references Ref1 and Ref2. Supposed we have known the distribution of T on Ref1 and the distribution of Ref1 on Ref2, there exist four types of relationships among all of gene fragments as Fig. 3 shows:

1. Case A: T matches Ref1 and Ref1 matches Ref2.
2. Case B: T mismatches Ref1 but Ref1 matches Ref2.
3. Case C: T matches Ref1 but Ref1 mismatches Ref2.
4. Case D: T mismatches Ref1 and Ref1 mismatches Ref2.

The above four cases are the most typical corresponding relationships between target sequence and two references. For case A, we should do match process, i.e. get and record the start position and end position of target sequence on Ref2 with the distribution of Ref1 on Ref2. For case B, C and D, we all do mismatch process, i.e. record the characters in this part, while in case C, we should use the partial decompression to achieve it.

According to the above-mentioned workflow, we put forward the simplest direct transformation algorithm, named TDM (transform by Direct Match). Its pseudocode is as the algorithm1 shows below. TDM just exploit distribution between Ref1 and Ref2.

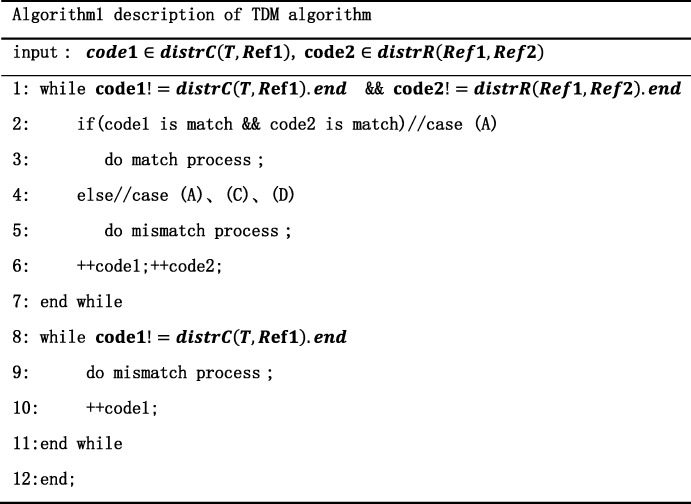



### TPI

**Fig. 4:**
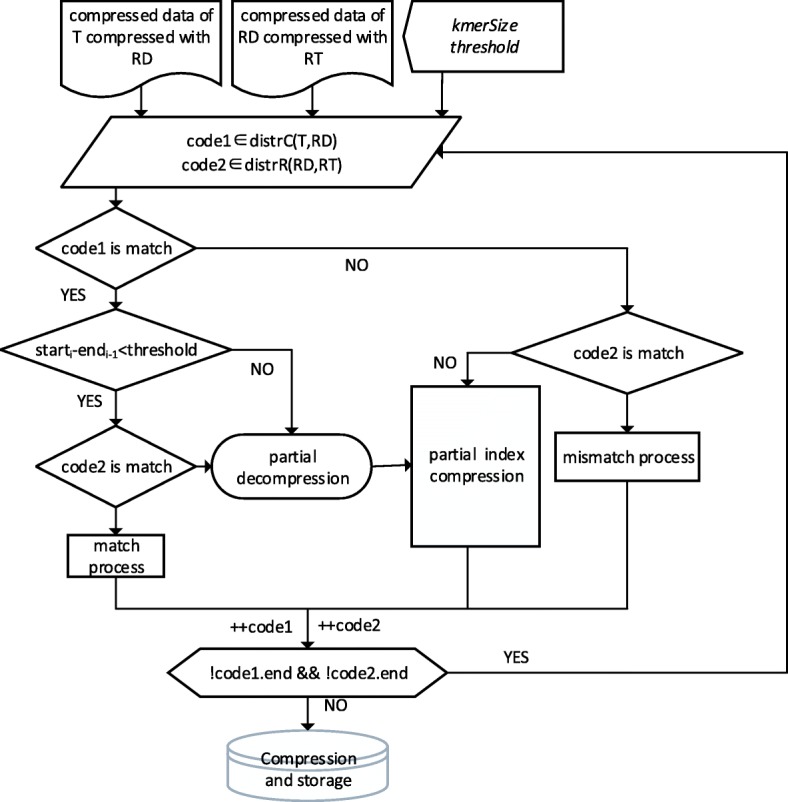
RD presents the reference the original dataset compressed with and the RT presents the reference we want transform RD to

As we can see, for case B, C and D in Fig. 4, TDM do mismatch process, resulting a decrease in compression ratio. To improve the compression ratio, we optimize this algorithm. According to the feature of encoding, we propose a more heuristic algorithm, named TPI (Transform by Partial Index). Its flowchart is as below.

As shown in the Fig. 4, TPI mainly optimize the mismatch process of TDM. For the four cases in the Fig. 3, we customize four different solutions:

1. In case A, if the length of match is short, doing match process will waste storage, so we should check whether it should do match process or mismatch process according to its length.
2. In case B, matches are not aligned. Such as slice alignment compression adapted by ERGC, when the start of target sequence can get longer match in the end of reference, it means that it is a misplaced match, so the characters in mismatch should do alignment and compression to improve the ratio.
3. In case C, like case B, for the target sequence, in the match, we should do partial decompression to get the characters and then do alignment and compression.
4. In case D, we can not get the relationship between T and Ref2, so for T and Ref2, in the mismatch, we should do small-range alignment and compression to improve the ratio.

Obviously, partial index compression algorithm is adopted in case B, C and D. The pseudocode of partial index compression algorithm is shown below.

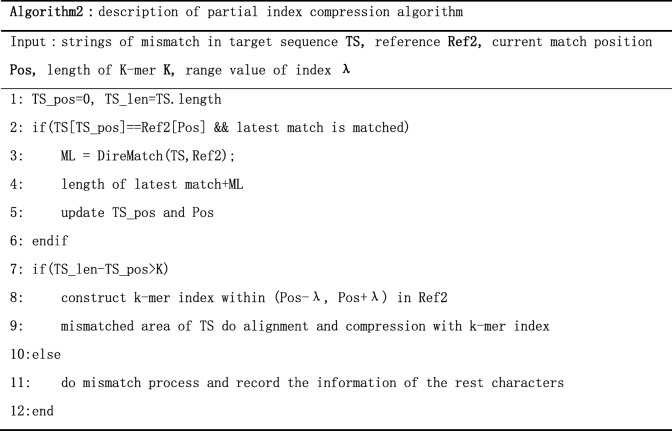



In algorithm2, we first extend the length of the latest match and then build the index of Ref2 within a certain range to do alignment and compression. This exploit the feature that genomes have high similarity at corresponding position. Generally, a match whose position is close to corresponding position is called a good match, because it need more space to encode the relative position if the relative is too far away. TPI can guarantee the relativity between the target sequence and reference by small-range alignment and compression.

### TGI

TDM and TPI are mainly designed for small-scale gene data transformation. Next, we will introduce an algorithm named TGI(Transform through Global Index) based on global index, which do transformation between gene banks faster at the price of taking more time to construct index.

Different with TPI, the input of TGI is two reference sequences and all compressed gene data in the gene bank.

TGI consists of 6 steps as below:

1. Construct the index of Ref2, which we will introduce later, and then get the distribution of Ref1 on Ref2 through aligning Ref1 with Ref2 using the index.
2. Pre-process the compressed gene data in gene bank to remove the effects of general compression, using general compression tool to obtain the intermediate compressed data. Then process the intermediate compressed data to get the distribution of target sequence T on Ref1.
3. Do alignment with the distribution of T on Ref1 and the distribution of Ref1 on Ref2 using the TGI matching algorithm, which will get more details later.
4. Return the match if what the step 3 return is match and the length of match is longer than the given threshold k. If the length of match is shorter than k, we decompress the segment and return the corresponding decompressed character information.
5. Do index alignment if step 3 or step 4 return the mismatch and the number of characters is more than k. If the number is less than k, we recorded the corresponding decompressed character information.
6. If step 4 return the match, we get the relative start position through start position of current matched area minus end position of previous match and get the length information through end position of current match minus start position of current match. Then record both. If this is the first match, we just need to get the length information. Return step 3.

Have known the framework of the algorithm TGI, we will get more details as follow.

### Construction of index

The index of Ref2 is hash table. Different with the strategy of dynamic memory allocation adopted by FRESCO, we pre-allocate the memory according to Mem(Index) ∝ Length(Ref2) to avoid reallocating the memory, copying the data and destroying the memory.

**Fig. 5:**
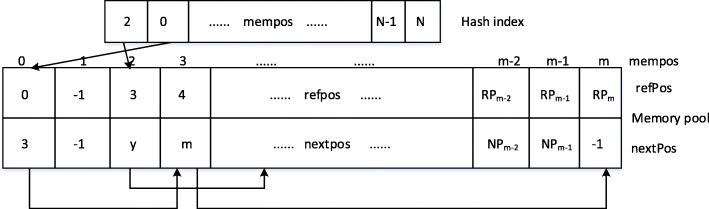
Index structure of memory pool

The Fig. 5 show the structure of the index. It is a one-dimensional array. Its index position is the hash key value and its value is the corresponding position in the memory pool. The entry in the memory pool is as below.

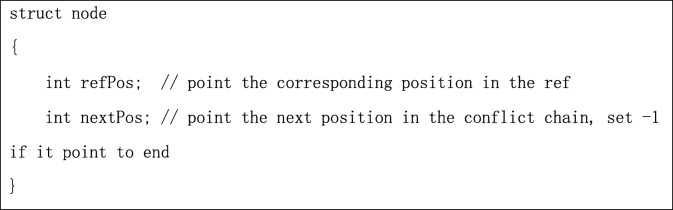



We can use the Rabin-Karp algorithm to void repetitive computation when we construct the index, which facilitate the similarity between the post k-mer subsequence and the previous k-mer subsequence.

### Match algorithm of TGI

**Fig. 6:**
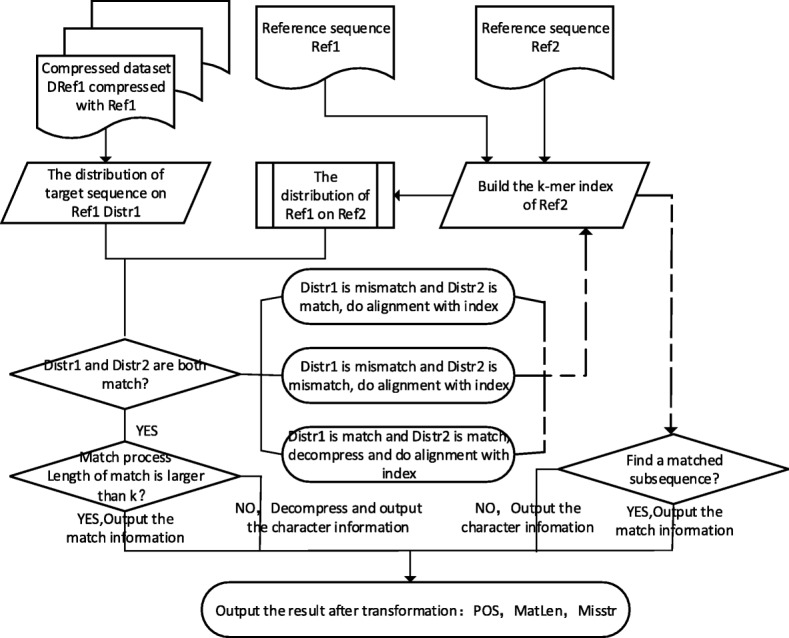
Flowchart of TGI

The essential part of the algorithm TGI is the match algorithm. As the Fig. 6 below shows, first, we should build the index of Ref2 and get the distribution of Ref1 on Ref2 through the index. There also are 4 cases like Fig. 3:

1. Case A: code1 is match and code2 is mismatch. If the length of the start position of code1 minus the end position of code1 is longer than the threshold, we take code1 as misplace match. Then we decompress the area, align and compress it with the index. If the length is shorter than the threshold, we take code1 as match. If the length of match is more than k, we record the start position and the length, if not, we record the character information of this area. The pseudocode of this part like Algorithm3.
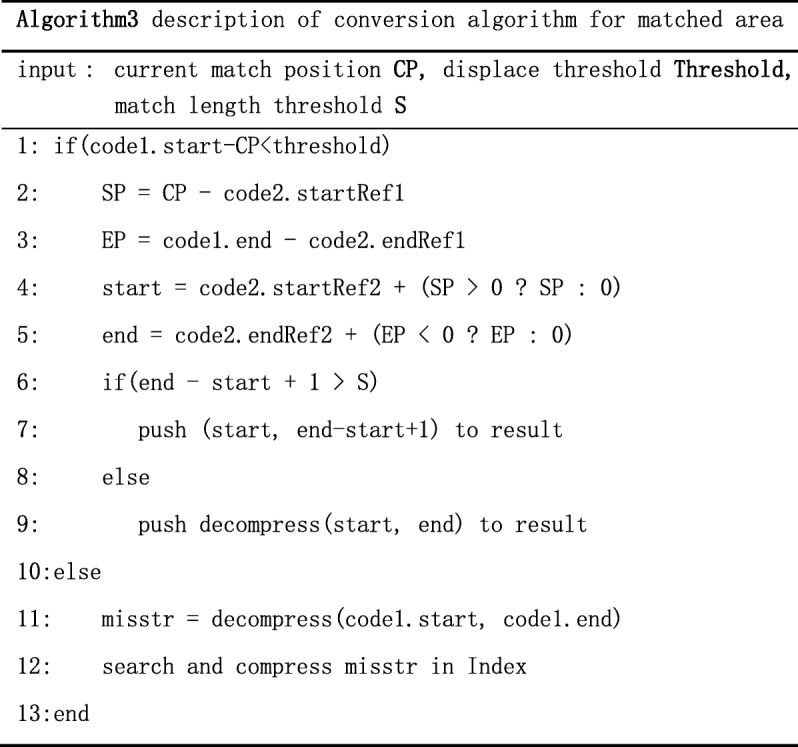

2. Case B. code1 is match and code2 is mismatch. If the match length is long, we can partially decompress and get the character information to enhance the compression ratio. Then we align and compress character information using the index of Ref2. If the matched length is short, we decompress this part directly and record the character information.
3. Case C: code1 is mismatch and code2 is match. Except the decompression, the process is same as case B.
4. Case D: code1 and code2 are both mismatch. If the previous transformation result is match, we align directly and compare characters of both area to extend the length of previous result. Then we check if we need to align and compress depending on the length of the rest part. If the previous transformation result is not match, we check if we need to align and compress depending on the length of the subsequence in Distr1. The pseudocode of this part like below:
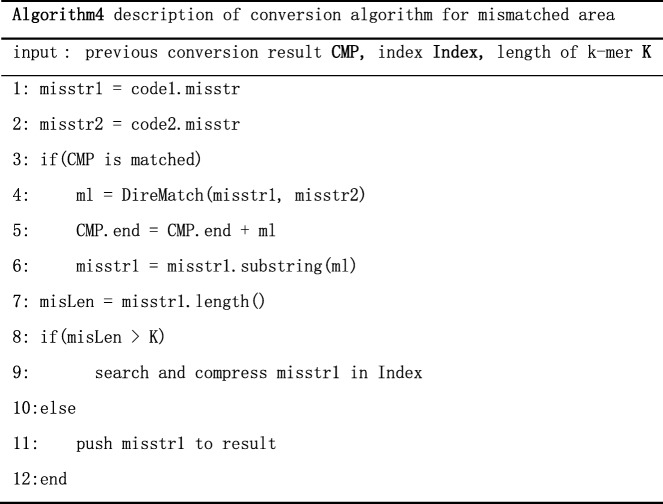

## Results

The test machine was two 2.50 GHz Intel Xeon E5–2680 v3 CPU with 64GB RAM running CentOS6.6 whose kernel version is 2.6.32 and GCC version is 4.4.7, JVM version is 1.6.0.

The dataset for test is same to the dataset used in ERGC. We selected the first Chinese standard genome sequence map YH-1 [28] sequenced by BGI, Korean gene data KOREF_200090131 (KOR131 for short) and KOREF_20090224 (KOR224 for short) [29]. All of these three datasets are human gene, which contain 24 chromosomes, each about 2990 MB in size. There exists a feature with these three datasets that they not only consist of {‘N’, ‘C’,‘A’,‘T’,‘G’}, but also contain some special characters like ‘U’,‘R’,‘S’,‘K’ and so on. ERGC can help us to handle all these special characters.

Next, we will use the datasets in the Table 1 below to do cross test for transformation algorithms, and then analyze our algorithms from three aspects of transformation time, compression ratio and memory consumption.

**Table 1:** Experiment datasets

dataset	Target sequence(Tar)	Reference 1(Ref1)	Reference 2(Ref2)
D1	YH-1	KOR131	KOR224
D2	YH-1	KOR224	KOR131
D3	KOR131	YH-1	KOR224
D4	KOR131	KOR224	YH-1
D5	KOR224	YH-1	KOR131
D6	KOR224	KOR131	YH-1

The main process of our test is getting the compressed data of Tar (as Table 1 shows, it means the target sequence) based on Ref2 from compressed data of Tar based on Ref1. The D1 in the Table 1 means that target sequence YH-1 based on KOR131 is transformed into compressed data based on KOR224. We can get the test result in Table 2 according to the experiment scheme in Table 1. The result is get by accumulating the processing time of all chromosomes and file size is the sum of all chromosomes.

**Table 2:** Result of transformation

ERGC			TDM		TPI		TGI		
dataset	Trans time	size	Trans time	size	Trans time	size	Trans time	size	Index time
D1	965.97	8.79	71.94	9.06	83.59	8.93	14.91	8.58	113.00
D2	989.05	8.97	71.77	9.12	119.67	9.06	14.85	8.50	113.25
D3	761.63	5.98	72.94	9.07	143.53	8.16	15.73	13.64	112.98
D4	847.25	13.05	72.08	13.28	84.20	12.86	8.26	8.89	99.69
D5	769.74	4.69	72.68	8.03	119.21	6.91	16.41	13.74	113.40
D6	824.82	11.57	72.07	12.04	129.52	11.44	8.25	9.07	102.32

**Fig. 7:**
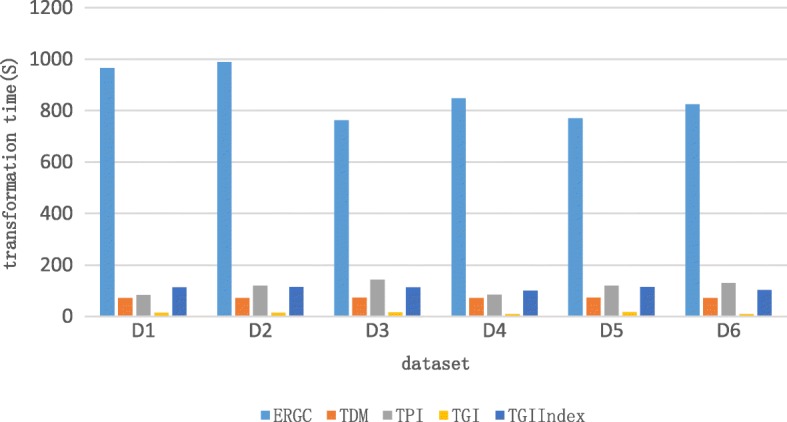
The unit of time is seconds and the transformation time of ERGC is the sum of decompression time of compressed data based on Ref1 and compression time of decompressed data based on Ref2

The main contribution of our new algorithms is simplifying the traditional workflow to attain the aim to reduce transformation time. As the Fig. 7 shows above, we can figure out that the transformation time of all three algorithms is an order of magnitude less than ERGC which adopts the traditional workflow. At the same time, it is obvious that TGI is much faster than TDM and TPI. This is because TGI constructs index for the reference. We also need to notice that building index is time-consuming.

Next, we will analyze the performances of these three algorithms on other indicators.

**Fig. 8:**
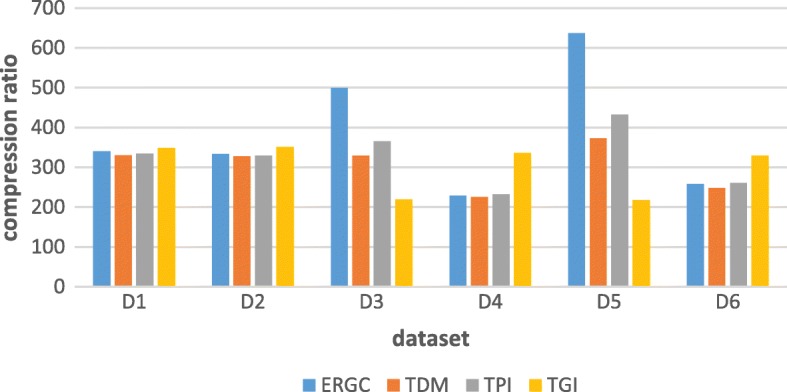
The original size of dataset is 2986.68 MB and the compression ratio presents like original data size: compressed data size

At first, let us pay attention on compression ratio. As Fig. 8 shows below, we can find that in dataset D1、D2、D4 and D6, the compression ratio of transformation algorithms are almost same to ERGC algorithm while TGI is a little higher than TDM and TPI. At the same time, in dataset D3 and D5, these three transformation algorithms, especially TGI, have some less in compression ratio. This is because dataset D3 is transformation from compressed KOR131 based on YH-1 to compressed KOR131 based on KOR224 and dataset D5 is transformation from compressed KOR224 based on YH-1 to compressed KOR224 based on KOR131. KOR131 and KOR224 are both Korean gene, the similarity between them is high than the similarity between them and Chinese gene. So, when the similarity between target sequence and Ref1 and the similarity between references is low, while the similarity between target sequence and Ref2 is high, our transformation will significantly reduce the compression ratio.

**Fig. 9:**
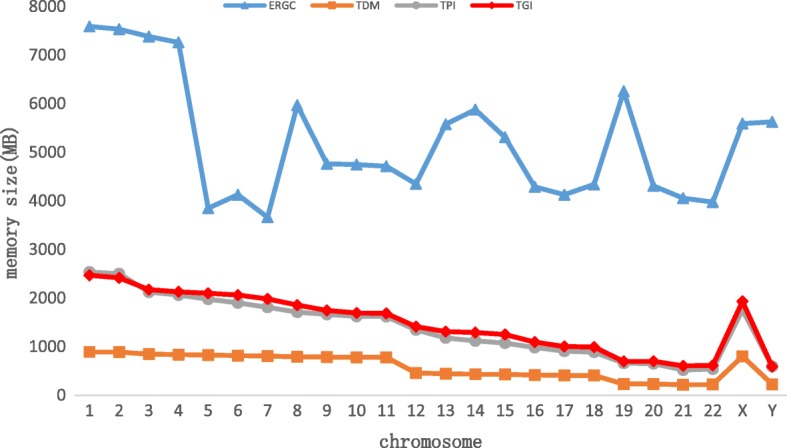
Memory consumption when running

Figure 9 below shows the comparison of the peak of memory consumed by the three algorithms when they are running. TDM just facilitate the similarity between references, so its memory consumption is low and it is positively related to the size of chromosome. TPI builds partial index, so its memory consumption will larger the than TDM. TGI is almost same to TPI. The memory consumption of ERGC is related to the compression of each fragment, so its memory consumption is unstable. At the same time, too much indexes make the memory consumption of ERGC is much more than the three transformation algorithms.

As we know, TGI need build index for reference. It can obviously reduce the transformation time, but it also cause expenses in other places. Next, we will analyze the efficiency of building hash index of references. In this experiment, we selected the gene dataset shown in Table 1 as reference. We compared the efficiency of building index by comparing FRESCO and TGI with the time of constructing index and the memory size of index. Since each dataset has 24 chromosomes, we selected chromosomes 1(236 MB), chromosomes X(148 MB), chromosomes 13(109 MB) and chromosomes 21(45 MB) from the YH dataset and chromosomes 3(189 MB) and chromosomes 28(75 MB) from the HG dataset by file size to analyze.

**Fig. 10:**
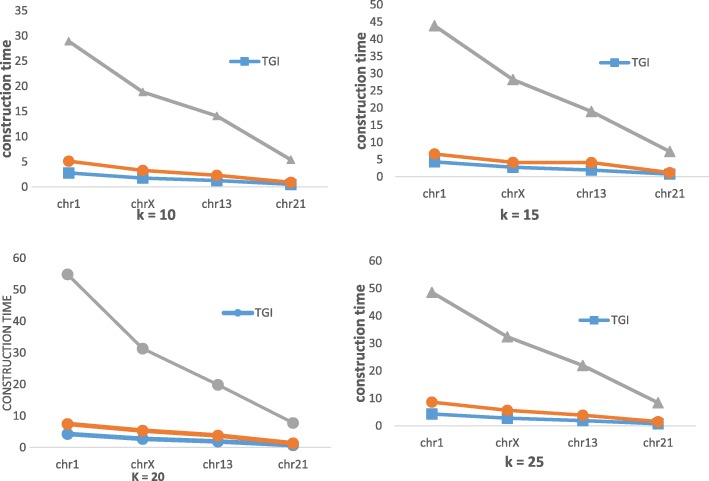
Time of constructing index at different values of k and different chromosomes

In Fig. 10, we compared the time of constructing index of 4 chromosomes from YH dataset at 4 different values of k. As we can see, the time significantly reduce after using a memory pool, for the dynamic allocation of memory is the most time-consuming. The time consumed by TGI combining the memory pool and the fast hash function is nearly twice the time consumed by the method using memory pool, and is 10 times less than the time consumed by FRESCO.

**Fig. 11:**
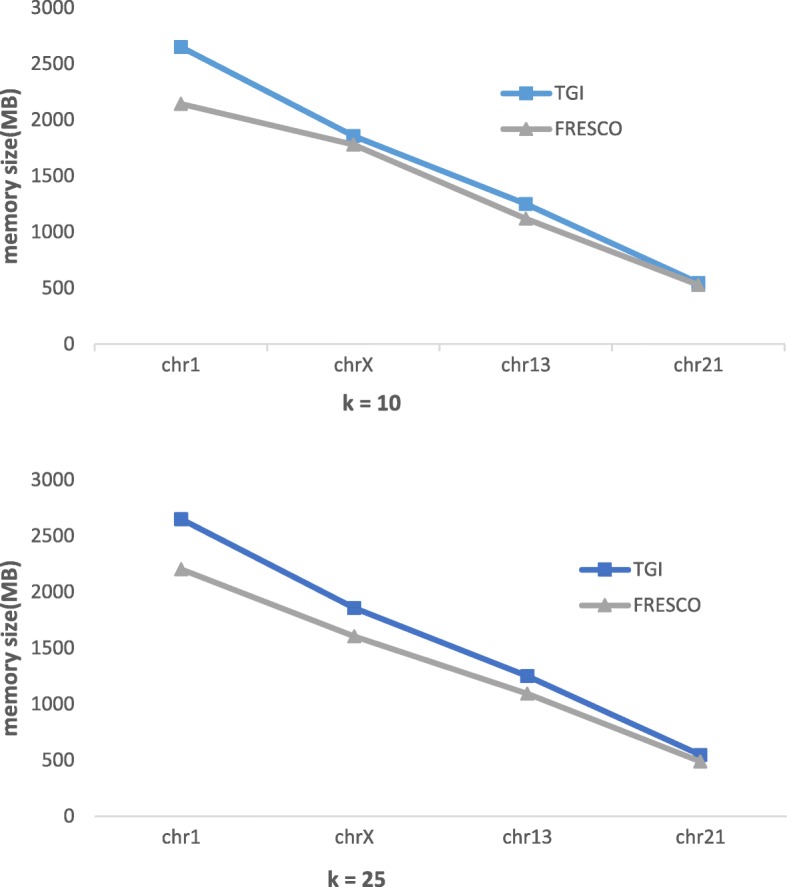
Memory size of index at different values of k

Because index structure of memory pool is same to index structure of TGI, we just compared memory consumption of TGI and FRESCO in Fig. 11. As we can see, the size of the index constructed by the two methods is positively related with size of the gene data. The size of index created by TGI using memory pool is larger than that by FRESCO using dynamic memory method, but doesn’t double.

## Discussion

We have introduced our three algorithms and the experiment results. There exists some points that we need to focus on.

Firstly, all of these are much faster than conventional decompression-and-compression method. These is the main attribution of this paper. We exploit the similarity between of references to reduce three steps to two steps. Secondly, as the most important indicator of compression algorithms, compression ratio of three new algorithms is almost same to the traditional algorithms. This is an important embodiment of them as good transformation methods. Finally, the transformation time of TGI is about 10 times faster than TDM and TPI, but the time of constructing index of TGI is much longer. Index just need to be constructed one time, so if there exists few data to transform, it is not worth wasting time to construct index. On the contrary, if there exist a large collection of gene data to transform, the construction time will be far less than the time we spend to align target sequence with reference. As a result, we can find that TDM and TPI are more suitable for small-scale gene transformation while TGI is more suitable for large-scale gene transformation.

As for the small-scale gene transformation, selecting TDM or TPI is also a question. TDM is a little faster than TPI and its memory consumption is less than TPI, but the compression ratio of TPI is better than TDM when the target dataset and destination dataset are not much similar. Our selection depend on what we care about.

## Conclusion

Trough discussion above, we can conclude that TDM and TPI are more suitable for small-scale gene data transformation and we select one of them depending on what we care about is transformation speed or compression ratio, while TGI is more suitable for large-scale gene data transformation.

Although the transformation speed of three algorithms we proposed is obviously faster than conventional decompression-and-recompression process, there are some aspects for optimization in the future.

1. Our algorithms mainly optimized the transformation time. Although we have adopted some methods to improve the compression ratio, there is still a certain loss in compression ratio. We can improve the compression ratio in the future.
2. In this paper, we just studied three compression tools, and the subsequent research can be done for more compression tools.
3. Due to the memory required of our algorithms is low, we can choose process pools or thread pools to improve the computation speed, or we can use the distributed file system speed up the IO by distributing the IO pressure on a single node to a number of nodes through a high-speed network.

## Abbreviation

k-mer: It is each motif of length k observed in a DNA sequence. Clearly, the number of k-mer in a sequence of length L we can obtain is L – k
